# Analyzing Femorotibial Cartilage Thickness Using Anatomically Standardized Maps: Reproducibility and Reference Data

**DOI:** 10.3390/jcm10030461

**Published:** 2021-01-26

**Authors:** Julien Favre, Hugo Babel, Alessandro Cavinato, Katerina Blazek, Brigitte M. Jolles, Thomas P. Andriacchi

**Affiliations:** 1 Swiss BioMotion Lab, Department of Musculoskeletal Medicine, Lausanne University Hospital and University of Lausanne (CHUV-UNIL), CH-1011 Lausanne, Switzerland; julien.favre@chuv.ch (J.F.); alessandro.cavinato1@gmail.com (A.C.); brigitte.jolles-haeberli@chuv.ch (B.M.J.); 2 Department of Mechanical Engineering, Stanford University, Stanford, CA 94305, USA; katerina.blazek@gmail.com (K.B.); tandriac@stanford.edu (T.P.A.); 3 Palo Alto VA, Palo Alto, CA 94304, USA; 4 Institute of Microengineering, Ecole Polytechnique Fédérale Lausanne (EPFL), CH-1015 Lausanne, Switzerland; 5 Department of Orthopaedic Surgery, Stanford University, Redwood City, CA 94061, USA

**Keywords:** cartilage, knee, morphology, osteoarthritis, registration, pattern

## Abstract

Alterations in cartilage thickness (CTh) are a hallmark of knee osteoarthritis, which remain difficult to characterize at high resolution, even with modern magnetic resonance imaging (MRI), due to a paucity of standardization tools. This study aimed to assess a computational anatomy method producing standardized two-dimensional femorotibial CTh maps. The method was assessed with twenty knees, processed following three common experimental scenarios. Cartilage thickness maps were obtained for the femorotibial cartilages by reconstructing bone and cartilage mesh models in tree-dimension, calculating three-dimensional CTh maps, and anatomically standardizing the maps. The intra-operator accuracy (median (interquartile range, IQR) of −0.006 (0.045) mm), precision (0.152 (0.070) mm), entropy (7.02 (0.71) and agreement (0.975 (0.020))) results suggested that the method is adequate to capture the spatial variations in CTh and compare knees at varying osteoarthritis stages. The lower inter-operator precision (0.496 (0.132) mm) and agreement (0.808 (0.108)) indicate a possible loss of sensitivity to detect differences in a setting with multiple operators. The results confirmed the promising potential of anatomically standardized maps, with the lower inter-operator reproducibility stressing the need to coordinate operators. This study also provided essential reference data and indications for future research using CTh maps.

## 1. Introduction

Knee osteoarthritis (OA) is a serious health concern [1,2], requiring better measure of the alterations in cartilage thickness (CTh) to allow more sensitive monitoring of its development and improve our understanding of its physiopathology. Magnetic resonance imaging (MRI) is the preferred modality for CTh measurement as it is three-dimensional (3D) and non-invasive [3,4]. MR images are usually segmented to create 3D CTh maps corresponding to the 3D surface of the subchondral bones, therefore describing CTh with high spatial resolution [5,6]. However, comparing CTh maps among different knees is challenging, because the size and shape of the subchondral bone surfaces differ from knee to knee. Consequently, CTh is usually quantified using mean values in predefined regions of interest (ROIs) [7,8,9,10]. While ROIs revealed to be useful to evaluate CTh and provided important insight into the pathogenesis of knee OA, their uses limit the analysis of spatial variations in CTh. In fact, CTh varies continuously (including within ROIs) and the variations have been shown to change with OA, possibly reflecting important features of the disease [11]. Therefore, there is a need for standardization methods to mitigate the differences in bone morphologies and allow for comparing maps between time points and knees.

Recently, a method was proposed to convert the 3D femoral and tibial CTh maps of any knee into 2D anatomically standardized CTh maps [12]. One promising aspect of this method compared to prior standardization methods [5,13] is that it uses anatomical correspondence to provide 2D “thickness images” that can be analyzed using common image processing techniques, therefore possibly improving the characterization of CTh. For example, anatomically standardized maps could be analyzed in terms of amplitude and location of particular CTh features [14,15] or in terms of spatial variations [15,16,17]. Nevertheless, before pushing such analyses, it remains necessary to assess the reproducibility of the 2D anatomically standardized CTh maps. The reproducibility assessment, combined with the technical validity of MRI-based CTh measurement [18], will provide the basis needed for the interpretation of CTh map data in the future. In accordance with literature, the reproducibility should be characterized for two regular experimental conditions, namely, when the data are processed by a single operator and when multiple operators participate in data processing [6,19,20,21].

Furthermore, since analyzing CTh using 2D anatomically standardized maps is a new approach, there is a need to quantify the differences among healthy knees in order to provide reference data for future uses of the standardization method. The study designs in the literature suggest that three comparison scenarios should be considered: when a knee is compared to itself, when a knee is compared to its contralateral knee and when a knee is compared to the knee of another individual matched for basic demographics [10,22,23,24,25].

This study aimed to provide a basis for the interpretation of 2D anatomically standardized CTh maps by characterizing the intra- and inter-operator reproducibility and quantifying the differences among healthy knees for three common comparison scenarios.

## 2. Materials and Methods

### 2.1. Experimental Procedure

Two groups of 10 healthy subjects each participated in this evaluation study. They were included on the basis of no history of serious lower-limb injury and absence of knee pain. The first group was composed of 10 convenience subjects (50% female; 24 ± 3 years old; 1.7 ± 0.1 m; 68 ± 11 kg), whereas the second group included 10 control subjects individually-matched to the subjects of the first group for gender, age (±4 years), height (±0.02 m) and weight (±3 kg). Similarly to previous CTh studies [6,26], their knees were imaged in the sagittal plane using a fat-saturated 3D spoiled gradient recalled echo sequence (3D-SPGR; TR = 60 ms; TE = 5 ms; flip angle = 40°; FOV 140 × 140 mm; in-plane resolution 256 × 256; slice thickness 1.5 mm; 60 slices) on a 1.5 T unit (GE Signa; GE Medical Systems, Milwaukee, WI, USA). The knees of the subjects in the first group were imaged twice, with the subjects exiting the MRI unit between scans. This study was approved by the Institutional Review Board and informed written consent was obtained from all subjects prior to data collection.

### 2.2. Anatomically Standardized Cartilage Thickness Map

**Figure 1:**
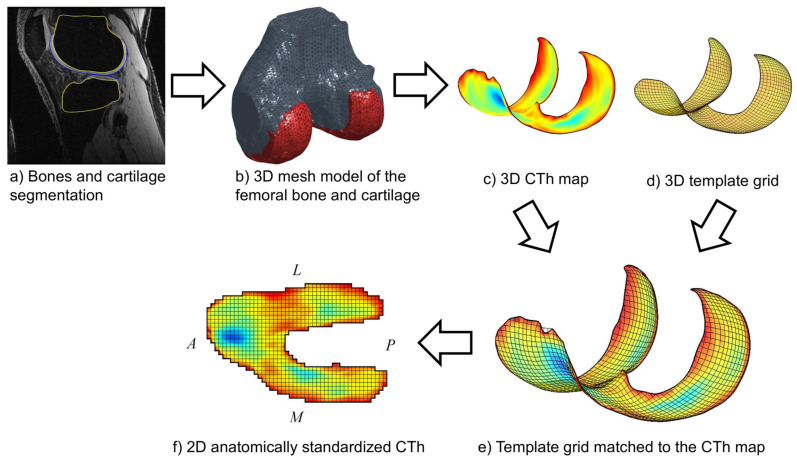
Illustration of the method (**a**–**f**) yielding the femoral 2D anatomically standardized CTh maps. The same method applies for the tibia. The A, P, L and M letters indicate the anterior, posterior, lateral and medial directions, respectively.

Anatomically standardized CTh maps were calculated for the femoral and tibial cartilages following a previously described procedure [12], summarized in this paragraph and illustrated for the femur in Figure 1. First, it required segmenting the bone and cartilage boundaries semi-manually on the MR images using custom software (Figure 1a), extending a previously published method limited to the segmentation of the cartilage [6]. The segmentation was performed by placing points at the bone and cartilage boundaries in order to create B-spline curves contouring these tissues [27]. Two operators segmented the MRI acquisitions independently. In order to get an estimation of the higher bound of inter-operator reproducibility, which is a safer approach to interpret the inter-operator reproducibility in the context of future studies, the two operators did not coordinate methods before segmentation [6]. Second, 3D mesh models of the femoral and tibial bone and cartilage tissues were reconstructed based on the segmentation data (Figure 1b). Third, the thickness of cartilage covering the bone was calculated, leading to 3D femoral and tibial subchondral CTh maps for each segmentation of each MRI acquisition (Figure 1c). Fourth, template grids of the subchondral bone areas (Figure 1d) were matched to the 3D femoral and tibial maps to establish anatomical correspondences based on the subchondral bone surfaces (Figure 1e). Fifth, the 3D femoral and tibial CTh maps were sampled according to the matched grids, resulting in 2D anatomically standardized CTh maps consisting of 5512 and 2063 pixels for the femur and the tibia, respectively (Figure 1f). All processing was done using custom-built software implemented in Matlab (Mathworks, Natick, MA, USA).

### 2.3. Statistical Analysis

The intra-operator reproducibility was assessed using 10 knees from 10 subjects that were imaged and processed twice by a single operator. The reproducibility was first quantified using Bland-Altman plots comparing the pixels of the 2D anatomically standardized CTh maps of the first and second imaging/processing [28]. Under the absence of heteroscedasticity in the Bland-Altman plots, the mean (*μ*) and standard deviation (*σ*) of the errors (i.e., differences between the first and second imaging/processing) can be defined as the reproducibility accuracy and precision. In this case, the accuracy quantifies the systematic errors (biases) for the entire maps, whereas the precision characterizes the variations of the errors throughout the maps. This assessment is however incomplete when 3D maps are transformed into 2D anatomically standardized maps because the transformation could distort the data, resulting in heterogeneous spatial distribution of the errors (i.e., the errors could be larger in some areas of the 2D map compared to others) that is not quantified by the accuracy and precision metrics. Therefore, second, to assess the spatial randomness of the errors, error maps were calculated as the pixel-by-pixel difference between the 2D anatomically standardized CTh maps of the first and second imaging/processing and the entropy was determined for each error map [29]. The error maps were stratified into 256 bins, leading to entropy values (*H*) between 0 (deterministic distribution indicating varying errors among areas) and 8 (uniform spatial distribution of the errors). Third, the reproducibility was assessed in terms of agreement between the 2D anatomically standardized CTh maps of the first and second imaging/processing. Quantifying the agreement is particularly relevant because one of the most promising aspects of 2D anatomically standardized maps is to allow the analysis of spatial variations in CTh. The agreement was evaluated using a two-way random-effects intraclass correlation coefficient (ICC) [30] between the pixels of the maps from the first and second imaging/processing. The accuracy, precision, entropy and agreement were first calculated separately for the femoral and tibial CTh maps. Since these metrics were not normally distributed among the 10 knees, they were summarized using the median and interquartile range (IQR). Two-sided Wilcoxon rank tests were done to compare the femoral and tibial reproducibility. When the results did not differ significantly between the femur and the tibia, the reproducibility metrics (accuracy, precision, entropy and agreement) were recalculated for the pixels of the femoral and tibial CTh maps together in order to get an overall assessment of the intra-operator reproducibility.

The inter-operator reproducibility was assessed using the same metrics (accuracy, precision, entropy and agreement) as described above. This time, the comparison was done for 10 MRI acquisitions processed by two operators: the 2D anatomically standardized CTh maps from the first operator were compared to the maps of the second operator. The intra- and inter-operator reproducibility values were compared using two-sided Wilcoxon rank tests.

To provide reference data regarding the differences that could be expected when healthy knees are compared using anatomically standardized maps, MRI acquisitions processed by a single operator were compared following three scenarios: (1) 10 knees compared to another acquisition of themselves (similar dataset as the intra-operator assessment); (2) 10 knees compared to their contralateral knees; and (3) 10 knees compared to the knees of 10 other persons individually matched for leg side, gender, height and weight. For the 30 comparisons (10 comparisons for each scenario), the differences between the pair of CTh maps were first quantified in terms of offset and dispersion calculated as the mean (*μ*) and standard deviation (*σ*) of the pixel-by-pixel differences between both maps. The differences were further characterized by the agreement (ICC) between the pixels of both maps. Kruskal–Wallis tests followed by two-sided Wilcoxon rank tests for post hoc analysis were used to compare the three metrics (offset, dispersion and agreement) among the three scenarios (same, contralateral and matched knees). All statistical analyses were done with MATLAB (Mathworks, Natick, MA, USA) using a significance level set *a priori* to *α* = 0.05.

## 3. Results

### 3.1. Intra- and Inter-Operator Reproducibility

**Figure 2:**
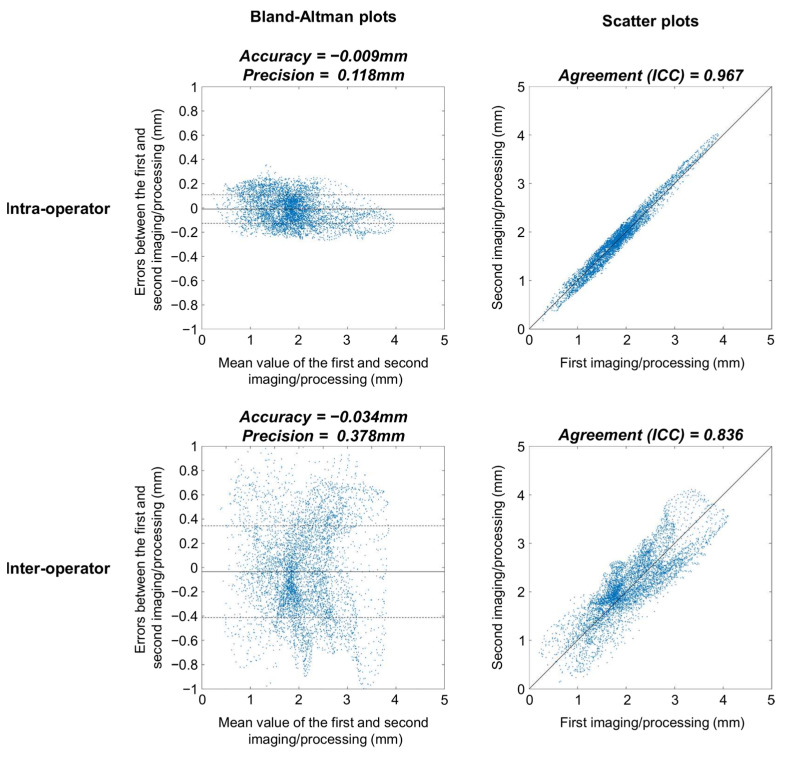
Bland-Altman (first column) and scatter (second column) plots of a typical femur for the intra-operator (first row) and inter-operator (second row) conditions. These four graphs are based on the 5512 pixels of the femoral 2D anatomically standardized map. In the Bland-Altman plots, the continuous lines represent the accuracy, while the dashed lines represent the precision. The black lines in the scatter plots correspond to the identity line.

**Figure 3:**
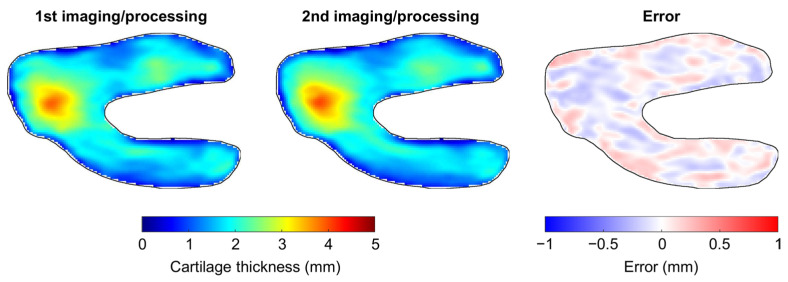
Illustration of the intra-operator reproducibility for a typical knee. This figure presents the anatomically standardized CTh map obtained with the first (left) and second (center) imaging/processing, as well as the error between the two maps (right).

None of the Bland-Altman plots for the assessment of intra-operator reproducibility showed heteroscedasticity, as illustrated for a typical femur in Figure 2 (upper left graph). There were no significant differences between femur and tibia reproducibility metrics (*p* ≥ 0.385) (Table 1). The overall (femoral and tibial maps together) median (IQR) intra-operator accuracy and precision were −0.006 (0.045) and 0.152 (0.070) mm, respectively. For comparison, the median femoral, tibial and overall CTh of the 10 knees were 1.95 (0.84), 2.15 (0.98) and 2.05 (0.75) mm. The overall median intra-operator entropy was 7.02 (0.71), indicating that the errors between the first and second imaging/processing were distributed heterogeneously. The spatial randomness of the errors between the first and second imaging/processing is illustrated in Figure 3. Finally, the overall median agreement between the first and second imaging/processing was 0.975 (0.020) (Table 1). This result is shown for a typical femur in the upper right graph of Figure 2.

Similar to the intra-operator condition, the Bland-Altman plots to assess the inter-operator reproducibility had no heteroscedasticity (see the lower left graph in Figure 2 for a typical example) and the inter-operator reproducibility metrics did not differ significantly between femur and tibia (*p* ≥ 0.076) (Table 1). The overall median inter-operator precision was 0.496 (0.132) mm, which was significantly higher than the intra-operator precision (*p* < 0.001). The inter-operator agreement (median of 0.808 and IQR of 0.108) was significantly lower than the intra-operator condition (*p* < 0.001). The differences in agreement between the intra- and inter-operator conditions are illustrated in the right column of Figure 2.

### 3.2. Differences among Healthy Knees

For the three comparison scenarios, the offsets between the pair of CTh maps were not significantly different whether femoral or tibial CTh was analyzed (*p* ≥ 0.089) (Table 2). Furthermore, there were no significant differences in offsets among comparison scenarios (*p* ≥ 0.212). The overall median values for the offsets were −0.006 (0.045), −0.036 (0.137) and 0.063 (0.254) mm for the comparison relative to the same knee, to the contralateral knee and to a matched knee, respectively.

The dispersion between the pair of CTh maps differed significantly between the femur and tibia for the matched knees comparison scenario, with lower dispersion values for the tibia (*p* = 0.003) (Table 2). The dispersions for the femoral cartilage were significantly lower for the comparison with the same knee than for the two other comparison scenarios and significantly lower for the comparison relative to the contralateral knee than for the comparison relative to a matched knee (*p* ≤ 0.017), with median values of 0.136 (0.085), 0.219 (0.092) and 0.456 (0.125) mm for the same, contralateral and matched knee comparison scenarios, respectively. Regarding the tibial cartilage, significantly lower dispersions were also observed for the comparison relative to the same knee than for both the comparison relative to the contralateral knee and the comparison relative to a matched knee (*p* ≤ 0.009), with median values of 0.146 (0.061), 0.241 (0.077) and 0.248 (0.051) mm for the same, contralateral and matched knee comparison scenarios, respectively.

The agreements between the pair of CTh maps were significantly different between the femur and the tibia for the matched knees comparison scenario, with higher agreement for the tibia (*p* = 0.003) (Table 2). Femoral agreements were significantly different among the three comparison scenarios (*p* ≤ 0.021). Specifically, the agreements for the comparison relative to the same knee (median of 0.974 and IQR of 0.017) were higher than for the comparison relative to the contralateral knee (median of 0.936 and IQR of 0.031), and the agreements for those two comparison scenarios were higher than for the comparison relative to a matched knee (median of 0.749 and IQR of 0.049). The agreements for the tibial maps were significantly higher for the comparison relative to the same knee than for the two other comparison scenarios (*p* ≤ 0.001), with median values of 0.976 (0.025), 0.936 (0.062) and 0.934 (0.033) for the same, contralateral and matched knee comparison scenarios, respectively.

## 4. Discussion

The reproducibility results confirmed the promising possibilities offered by 2D anatomically standardized maps to analyze CTh. The high entropy and agreement values obtained when the knees were imaged and processed twice by a single operator (intra-operator reproducibility) indicated that the maps were slightly distorted between imaging/processing. In addition, the low accuracy values indicated that there was barely no systematic error between imaging/processing, which is consistent with previous studies [19,31]. This absence of bias is a critical result in support of CTh comparison through maps because it suggests that the imaging/processing operations are unlikely to lead to false detection of CTh differences among maps. The precision results, on the other hand, provided information on the random noise affecting the maps. The median precision of 0.15 mm was about 14 times less than the median CTh of the test knees, suggesting that the precision is adequate to capture the spatial variations in CTh. This observation agrees with the high agreement values, showing that the spatial variations were extremely similar between imaging/processing. While comparing the reproducibility of the 2D anatomically standardized CTh maps with prior MRI literature is difficult because different metrics apply to maps and mean CTh measure in regions of interest (ROIs), a comparison could be attempted regarding the precision. In this regard, the 0.15 mm obtained in the present study well agrees with prior works where precision is reported to vary between 0.04 and 0.30 mm [6,19,20]. It is worth noting that this comparison is indicative as many elements differ among studies, including the imaging protocols and the characteristics of the knees. Additionally, as the precision is related to the sensitivity to detect CTh differences among knees, it is relevant contrasting this value to the differences in CTh previously reported with knee OA. Doing so further suggests that maps are adequate as the 0.15 mm of precision is less than the statistically significant differences of 0.2 to 0.5 mm reported between non-OA and early OA knees, and less than the 0.3 to 1.8 mm reported between non-OA and severe OA knees [12,23]. These comparisons suggest that CTh maps should be able to detect the differences among knees at varying OA stages. In summary, the entropy, agreement, accuracy and precision results all indicated a suitable intra-operator reproducibility of CTh maps, supporting their use with knee OA, particularly to describe and compare spatial variations in CTh.

Characterizing the differences among anatomically standardized CTh maps of healthy knees for three common study scenarios provided reference data and indications that will be useful to interpret the results of future research. The absence of significant differences among the offset metric for the three comparison scenarios suggested that the differences in average CTh over the entire femoral and/or tibial articular surfaces are similar, whether the knees are compared to themselves, to their contralateral knees or to the knees of matched individuals. While this observation could be seen as positive because it indicates that the selection of the comparison knees (same, contralateral or matched) has little effect, it rather underlines the low sensitivity of average measures to detect CTh differences among knees [32]. In contrast to the offset, the dispersion and agreement results differed among comparison scenarios. For the femur, the similarity of spatial variations in CTh between maps continuously decreased from comparisons to the same knee, to comparisons to the contralateral knee and comparisons to a matched knee. A decrease was also observed for the tibia, with the same knee scenario reporting higher similarity than the two other scenarios. While spatial variations in CTh were marginally studied in the past due to the paucity of means to quantify them, these findings agree well with the literature. In fact, healthy articular cartilage has been shown to be adapted to its mechanical environment, which for the knee joint is primarily conditioned by the cyclic loading during walking [33,34]. Regarding femoral cartilage, prior research has specifically reported the amplitude of CTh to be associated with the knee adduction moment, a surrogate variable of loading amplitude [26,35], and the location of thicker cartilage to be associated with the knee flexion angle, a surrogate variable of loading location [14,36]. Therefore, under the assumption that the two knees of a single healthy individual are exposed to more similar loading patterns than two knees from matched individuals [37,38], it makes sense that the dispersion and agreement results indicated higher similarity in femoral CTh spatial variations for the contralateral comparisons than for the matched comparisons. Then, regarding tibial CTh, prior research has reported an association between the knee adduction moment and CTh amplitude, but not between the knee flexion angle and the location of thicker cartilage [35,36]. This suggests that the individual loading patterns have less effect on the spatial variations in CTh for the tibia than for the femur. This also well agrees with the present results as no differences were observed between contralateral and matched comparison scenarios for the tibial cartilage. Therefore, in addition to providing quantitative reference data to help interpreting differences among CTh maps in future studies, these results further stress the potential value of analyzing the spatial variations in CTh [32]. In agreement with literature, the spatial variations in CTh were shown to be more subject-specific on the femur than on the tibia [35], suggesting that the decision of testing femoral and/or tibial cartilages in future studies could depend on the specific objectives and design of these future works.

Literature consistently reported lower reproducibility of mean CTh measure in ROIs when the MRI processing was done by different operators than when a single operator processed the data multiple times [6,19,20,21]. Analyzing CTh using 2D anatomically standardized maps was no different as the reproducibility was generally lower with the inter- than intra-operator condition. The accuracy and entropy values did not differ significantly between conditions, suggesting that the systematic error and the spatial randomness of the error were comparable between the two experimental conditions. These observations are encouraging because they suggest that false detection of CTh differences among maps should be limited when the processing is shared between operators. On the other hand, the precision values were about three times higher when two operators processed the MRI than when a single operator processed the MRI twice. The inter-operator precision errors corresponded to about a fourth of the median CTh of the test knees, which was not negligible. A consequence of these relatively high precision values was to blur the CTh maps, as shown by the lower agreement values for the inter- compared to intra-operator conditions. These observations suggest a possible loss of sensitivity to detect CTh differences among maps when the MRI processing is shared between operators. However, it is difficult to estimate the concrete effects the higher precision and lower agreement values in the inter-operator condition will have on CTh maps analysis. In fact, 2D anatomically standardized CTh maps are new and there are not enough data in literature to provide a tangible estimation of the effects of the increased precision and decreased agreement values in the inter-operator condition. In line with this comment, it is worth mentioning that the inter-operator values should also be interpreted carefully as they are based on two specific operators and that the reproducibility could be different between other operators. Nevertheless, to limit this influence, the two operators in this study did not coordinate methods before segmenting the MRI. With this approach, the results in Table 1 should correspond to an estimation of the higher bound of inter-operator reproducibility, which provide a safer characterization in the perspective of helping the interpretation of future map data. Nevertheless, while it seems clear that having all the MRI processed by a single operator is preferable, the inter-operator reproducibility could certainly be improved if the operators use similar segmentation guidelines (i.e., follow a common training) or if a quality check or even a final adjustment of the segmentation is performed by a single individual [6,21]. Additionally, when the processing has to be shared between several operators, it might be better having some operators only segmenting the femoral cartilage and the others only segmenting the tibial cartilage. This way, the number of operators contributing to the processing of each cartilage will be less, which should improve the inter-operator coordination.

This study has some limitations that are worth discussing. First, the results were obtained based on two operators analyzing a small number of healthy knees. The numbers of operators and knees were nonetheless consistent with the objectives of the study and comparable to prior reproducibility studies [18,39]. The two operators involved in this study were regular members of our groups participating in research about knee morphology. They were selected based on time availability and should therefore represent usual operators. Although repeating the analyses with knees affected by particular pathologies could be necessary in the framework of future research, literature suggests that the present results should remain similar with pathological knees [20]. In particular, a strength of the anatomical standardization used in this study is that it relies only on the subchondral bone areas. Therefore, while diverse factors, such as gender, mechanical alignment, bone remodeling and osteophytes, could influence the overall shape of the distal femur or proximal tibia, their impact on the current standardization method should remain limited. Nevertheless, future studies using 2D anatomically standardized CTh maps should bear in mind that the spatial variations in CTh could be influenced by various factors and that these factors might need to be included as confounding variables in the statistical analyses. Second, while the reproducibility and comparison analyses reported in this work are critical to understand the potential of 2D anatomically standardized CTh maps and to use them appropriately in future studies, one should keep in mind that the present characterization informs about the comparison of individual pairs of maps pixel-by-pixel. Future research using CTh maps are expected to analyze the maps using image processing techniques, such as statistical parametric mapping [17], texture analysis [16] or neural network classification [40]. Generally, these techniques do not consider images as series of independent pixels but rather as arrays of related pixels. Consequently, these techniques should be more sensitive and robust to differences in CTh maps than suggested by the reproducibility results in the present study. Third, while measuring CTh using 1.5 T MRI with in-plane resolution of 0.55 mm^2^ was repeatedly shown to be valid [6,26,41], it is probable that the intra- and inter-operator reproducibility would improve with higher magnetic field and in-plane resolution. Interestingly, both the intra- and inter-operator precision errors were less than the in-plane MRI resolution, which further supports the suitability of CTh maps. Finally, bones and cartilages were segmented semi-manually, as it is usually done nowadays. The method to calculate 2D anatomically standardized CTh maps is however compatible with any segmentation approach and enhanced reproducibility could be obtained in the future with automatic segmentation [42,43]. Interestingly, although this study used sagittal-plane images, which increase the difficulty to segment the medio-lateral edges of the femoral condyles because they are parallel to the acquisition plane, the errors remain homogeneously distributed over the femoral maps. Indirectly, this observation constitutes another evidence for the robustness of the anatomically standardized CTh maps.

In conclusion, this study showed that 2D anatomically standardized CTh maps are reproducible, confirming their promising potential, particularly for the analysis of spatial variations in CTh. As expected, the reproducibility was lower when two operators processed the MRI, which supports the need to coordinate operators. This work also provided essential reference data and indications for future research using CTh maps. It notably showed that the subject-specificity of the spatial variations in CTh were different for the femur and the tibia, indicating that future studies might prefer focusing on the femoral or tibial cartilages depending on their research questions and study designs. In this study aiming at evaluating the standardization method, CTh was reported in millimeters. However, future applications could work with normalized CTh data, for example using z-scores [44] or ratios [41]. Mathematically, CTh maps are nothing but images, that could be analyzed with numerous image processing techniques. Consequently, they could lead to multiple new ways to analyze CTh, possibly enhancing the detection of CTh differences in research projects and allowing more personalized management of patients with knee OA. In addition, anatomically standardized maps could be extended to analyze other tissue properties, such as cartilage and subchondral bone texture [45,46] or cartilage composition [47], which could provide complementary information to CTh in understanding the healthy knee joint and characterizing its pathologies.

## Figures and Tables

**Table 1:** Intra- and Inter-Operator Reproducibility.

	Intra-Operator		Inter-Operator	
	Median	IQR	Median	IQR
Accuracy (*µ*), mm				
Femur	−0.002	0.059	−0.056	0.196
Tibia	0.005	0.054	−0.098	0.616
Femur + tibia	−0.006	0.045	−0.003	0.186
				
Precision (*σ*), mm				
Femur *	0.136	0.085	0.371	0.131
Tibia *	0.146	0.061	0.467	0.258
Femur + tibia *	0.152	0.070	0.496	0.132
				
Entropy (*H*)				
Femur	7.27	0.65	7.26	0.56
Tibia	7.13	0.66	7.17	0.39
Femur + tibia	7.02	0.71	6.92	0.32
				
Agreement (*ICC*)				
Femur *	0.974	0.017	0.823	0.094
Tibia *	0.976	0.025	0.769	0.145
Femur + tibia *	0.975	0.020	0.808	0.108

Data are presented as median and interquartile range (IQR). The stars (*) indicate significant differences between the intra- and inter-observer conditions (*p* < 0.001).

**Table 2:** Differences among 2D Anatomically Standardized CTh Maps for Three Comparison Scenarios.

	Differences Compared to					
	Same Knee		Contralateral Knee		Matched Knee	
	Median	IQR	Median	IQR	Median	IQR
Offset (*µ*), mm						
Femur	−0.002	0.059	−0.033	0.107	0.064	0.261
Tibia	0.005	0.054	−0.086	0.180	0.103	0.215
Femur + tibia	−0.006	0.045	−0.036	0.137	0.063	0.254
						
Dispersion (*σ*), mm						
Femur *^,#,¶^	0.136	0.085	0.219	0.092	0.458	0.125
Tibia *^,#^	0.146	0.061	0.241	0.077	0.248	0.051
Femur + tibia *	0.152	0.070	0.233	0.074	Ø	
						
Agreement (*ICC*)						
Femur *^,#,¶^	0.974	0.017	0.936	0.031	0.749	0.049
Tibia *^,#^	0.976	0.025	0.936	0.062	0.934	0.033
Femur + tibia *	0.975	0.020	0.936	0.020	Ø	

Data are presented as median and interquartile range (IQR). The empty symbols (Ø) indicate significantly different results for the femur and tibia (*p* ≤ 0.003). In the cases of varying results between tibia and femur, no overall assessment was performed. Symbols indicate differences between study scenarios that achieved statistical significance (*p* < 0.05): * between comparison to the same knee and comparison to the contralateral knee; ^#^ between comparison to the same knee and comparison to a matched knee; ^¶^ between comparison to the contralateral knee and comparison to a matched knee.

## Data Availability

The data are not publicly available due to regulatory provisions.
